# CT versus FDG-PET/CT response evaluation in patients with metastatic colorectal cancer treated with irinotecan and cetuximab

**DOI:** 10.1002/cam4.271

**Published:** 2014-06-18

**Authors:** Kristin Skougaard, Helle Hjorth Johannesen, Dorte Nielsen, Jakob Vasehus Schou, Benny Vittrup Jensen, Estrid V S Høgdall, Helle Westergren Hendel

**Affiliations:** 1 Department of Oncology, Copenhagen University Hospital HerlevHerlev, Denmark; 2 Department of Radiology, Copenhagen University Hospital RigshospitaletHerlev, Denmark; 3 Department of Pathology, Copenhagen University Hospital HerlevHerlev, Denmark; 4 Department of Nuclear Medicine, Copenhagen University Hospital HerlevHerlev, Denmark

**Keywords:** Colorectal cancer, *KRAS*, PERCIST, PET/CT, RECIST, response evaluation, survival

## Abstract

We compared morphologic computed tomography (CT)-based to metabolic fluoro-deoxy-glucose (FDG) positron emission tomography (PET)/CT-based response evaluation in patients with metastatic colorectal cancer and correlated the findings with survival and *KRAS* status. From 2006 to 2009, patients were included in a phase II trial and treated with cetuximab and irinotecan every second week. They underwent FDG-PET/CT examination at baseline and after every fourth treatment cycle. Response evaluation was performed prospectively according to Response Evaluation Criteria in Solid Tumors (RECIST 1.0) and retrospectively according to Positron Emission Tomography Response Criteria in Solid Tumors (PERCIST). Best overall responses were registered. Sixty-one patients were eligible for response evaluation. Partial response (PR) rate was 18%, stable disease (SD) rate 64%, and progressive disease (PD) rate 18%. Partial metabolic response (PMR) rate was 56%, stable metabolic disease rate 33%, and progressive metabolic disease (PMD) rate 11%. Response agreement was poor, *κ*-coefficient 0.19. Hazard ratio for overall survival for responders (PR/PMR) versus nonresponders (PD/PMD) was higher for CT- than for FDG-PET/CT evaluation. Within patients with *KRAS* mutations, none had PR but 44% had PMR. In conclusion, morphologic and metabolic response agreement was poor primarily because a large part of the patients shifted from SD with CT evaluation to PMR when evaluated with FDG-PET/CT. Furthermore, a larger fraction of the patients with *KRAS* mutations had a metabolic treatment response.

## Introduction

Computed tomography (CT) is still the most frequently used imaging modality for response assessment of patients with metastatic colorectal cancer (mCRC) 1,2 and Response Evaluation Criteria in Solid Tumors (RECIST 1.0) 3 are the most frequently applied criteria. Also, assessing treatment response with fluorine-18 fluoro-deoxy-glucose-positron emission tomography/CT (FDG-PET/CT) has shown to be resourceful for both response prediction and evaluation of cancer patients 4–8 and to correlate well with survival 9,10 in spite of greatly different outcomes, including higher response rates (RRs), than seen with CT 11–13. This tendency is seen with chemotherapy and furthermore with targeted therapies 8,11,14 suggesting that in this setting, visualization of metabolism could be a suitable mean for response assessment 14–16. In clinical phase II trials, patients with complete response (CR), partial response (PR), and stable disease (SD) will continue treatment until progression is verified by CT. Yet, it is the response rate (RR = fraction of patients with CR and PR) that determines whether a tested drug or regimen will be further investigated in a phase III trial or implemented as standard treatment. Low RRs from CT-based trials could lead to efficacy underestimation of potentially active targeted treatments and maybe in withdrawal rather than further testing 14,15. Choosing a response evaluation modality and method that, as precisely as possible, reflects the investigated drug's mode of action is, therefore, important in order to present accurate and applicable RRs.

Approximately 35% of patients with CRC harbor a codon 12/13 mutation in the cancer cell proto-oncogene *KRAS*. Studies, using CT-based response evaluation, have shown that *KRAS* mutation is a negative predictive marker of morphologic response to the monoclonal antibody cetuximab 17,18. The effect of cetuximab on tumor metabolism, visualized by FDG-PET/CT, has not been investigated in patients with mCRC harboring *KRAS* mutations. Applying both morphologic and metabolic response visualization, as done in the present investigation, will elucidate the differences between the two methods and clarify how FDG-PET/CT response evaluation and *KRAS* mutation status is correlated.

In this study, we compared CT response evaluation with RECIST 1.0 to FDG-PET/CT response evaluation with Positron Emission Tomography Response Criteria in Solid Tumors (PERCIST) 19 and correlated the findings to overall survival (OS) and *KRAS* status.

## Patients and Methods

### Patients

From 2006 to 2009, patients with mCRC were, regardless of *KRAS* mutation status, prospectively included in a phase II trial and treated every second week with a combination of the epidermal growth factor receptor (EGFR) specific monoclonal antibody cetuximab (Erbitux®; Merck, Darmstadt, Germany; 500 mg/m²) and the chemotherapeutic drug irinotecan (Irinotecan; Fresenius Kabi Oncology, Bad Homburg, Germany; 180 mg/m²) as 3rd line palliative treatment. The protocol was approved by the Danish Regional Research Ethics Committee, The Danish Medicines Agency (EudraCT nr. 2006-001961-40), and The Data Protection Agency. Oral and written informed consent was obtained from all patients before inclusion.

The patients were scanned between 1 and 14 days prior to the first treatment and after every fourth treatment cycle until progression was established according to RECIST. Prednisolone was only administered to the patients on the day of treatment and the two following days.

### FDG-PET/CT examinations

The patients were examined from base of scull to mid-thigh on one of two different scanners: Philips Gemini Dual Slice PET/CT (Gemini DS) or Philips Gemini TruFlight™ (TF) 16-slice PET/CT (Gemini TF) (Philips Medical Systems, Cleveland, OH). Philips Extended Brilliance Workspace Nuclear Medicine version 2.0, Tumor Tracking was used to draw regions of interest (ROIs). The applied tracer was 18 F-FDG. It was injected intravenously (i.v.) with an aimed dose of 370 ± 10% MBq. The patients fasted ≥5 h before scan start. Blood glucose was measured immediately before tracer injection and patients with levels ≥8 mmol/L were excluded. Uptake time from tracer injection to onset of emission scan was aimed at 60 ± 10% min. The multidetector spiral CT scans were standard diagnostic contrast-enhanced examinations covering the thorax, abdomen, and pelvis. Iodinated contrast agent (Omnipaque 350; GE Healthcare, Oslo, Norway) was given orally: 20 mL in 500 mL bottled water (4% solution) half an hour before scan start, and i.v.: 100 mL with an injection flow of 5 mL/sec immediately before scan start.

It was intended to examine each patient on the same scanner throughout their treatment course. Patients who were examined on the two scanners in a manner precluding response evaluation were excluded, whereas patients with one or few examinations performed on the scanner different from their baseline-scanner were included if elimination of the irregular examinations was possible without affecting response evaluation.

The PET and the CT scan were described separately in the Department of Nuclear Medicine and in the Department of Radiology, respectively. Thereafter, a joint conclusion, containing both convergent and divergent findings, was performed in collaboration between the nuclear physiologist and the radiologist. Neither were blinded to previous scans and both had in principal access to their opposite scans. Continuation or termination of treatment was based on the prospective CT response evaluation only. PET evaluation was performed retrospectively.

### Response evaluation with RECIST

Target lesions, up to five per organ and 10 in total, were chosen on the baseline CT, measured in the longest diameter and the diameters were summed. On each subsequent examination, the target lesions were measured and summed. Nontarget lesions were registered at baseline and described on each subsequent examination. Response was calculated as Δ∑longest diameter between baseline and actual follow-up divided by baseline ∑longest diameter × 100%. If ∑longest diameter increased, response was calculated as ∑longest diameter between lowest registered and actual ∑longest diameter divided by lowest registered ∑longest diameter × 100%.

Responses were, according to RECIST 3, categorized in CR, PR, SD, and progressive disease (PD). The best overall morphologic response (BOR) achieved by each patient during treatment was registered. RR, clinical benefit rate (CBR = fraction of patients with CR, PR and SD ≥ 6 months) and disease control rate (DCR = fraction of patients with CR, PR, and SD) were calculated.

### Response evaluation with PERCIST

FDG-uptake was normalized to lean body mass (lbm) and termed standard uptake value-lbm (SUL). Background and lesion ROIs were drawn according to the guidelines 19. In the hottest (= highest FDG-uptake) part of the hottest lesion, a 1.2-cm-diameter spherical ROI (∼1 cm^3^) was drawn. The ROI was placed where it resulted in the highest possible SULmean value = SULpeak. The hottest lesion during follow-up could be a lesion different from the previously measured; presupposing it had been present since baseline. If baseline SULpeak in the single hottest lesion did not exceed the defined background value, the patient was not eligible for response evaluation. Response was calculated as ΔSULpeak between baseline and actual follow-up divided by baseline SULpeak × 100%. If SULpeak increased, response was calculated as ΔSULpeak between lowest registered SULpeak and actual SULpeak divided by lowest registered SULpeak × 100%.

Responses were categorized according to PERCIST 19 in complete metabolic response (CMR), partial metabolic response (PMR), stable metabolic disease (SMD), and progressive metabolic disease (PMD). Best overall metabolic response (BOMR) achieved by each patient during treatment was registered and metabolic response rate (MRR = fraction of patients with CMR and PMR) was calculated.

### *KRAS* analysis

Formalin-fixed paraffin-embedded tumor tissue, collected at the time of diagnosis, was used to evaluate and confirm the presence of tumor tissue. Verification of tumor cells was done by hematoxylin and eosin staining. DNA extracted from three sections was subjected to Therascreen® KRAS real-time PCR assays (DxS Ltd, Manchester, U.K.), which identified seven mutations in codon 12 and codon 13 using an ABI7500 real-time PCR platform. The patients were classified as harboring *KRAS* mutations if one of the seven mutations were present or as *KRAS* wild-type if no mutations were present 20,21.

### Statistical analysis

The kappa statistic was used for agreement analysis. The Kaplan–Meier method was used for OS analysis with the log-rank test for *P*-value calculation and Cox-regression analyses for hazard ratio (HR) and confidence interval (CI) calculations. OS was defined as time from trial registration of a patient until death of any course.

## Results

### Patients

Among 150 included patients, 131 were examined with FDG-PET/CT during their treatment course. Of these, 13 patients were never scanned and never treated and 37 were excluded due to their own wish, anaphylactic reactions to the first treatment or clinical progression before first follow-up. One patient with bg ≥ 8, one with no measurable disease on PET, and four with unavailable PET images were excluded. Furthermore, two patients without target lesions according to PERCIST and 12 that were examined on the two different scanners in a manner preventing response evaluation were excluded. Ultimately, 61 patients were eligible for response evaluation with CT and FDG-PET/CT. Characteristics are given in Table 1.

**Table 1:** Characteristics of the 61 evaluated patients

Characteristics	Value
Age (years)	
Median	62
Range	36–82
Sex (number of patients)	
Female	24
Male	37
Weight (kg)	
Median	77
Range	49–118
Number of metastatic sites	
Median	2
Range	1–4
*KRAS* status	
Wild-type	42 (69%)
Mutations	18 (30%)
Unknown	1 (∼1%)
Number of treatments per patient	
Median	8
Range	4–28
Number of examinations	
Total	203
Median	3
Range	2–7
Scanner (number of patients)	
Philips Gemini dual slice	56
Philips TF 16 slice	5
Treatment received after progression to the protocol-treatment (irinotecan + cetuximab)	
No treatment	30
Cetuximab + irinotecan + sunitinib	23
Cetuximab + irinotecan + bevazicumab	3
Other	5

Of the total 230 examinations, 27 were not performed on the scanner the individual patient was examined in at baseline and were, therefore, excluded; leading to 203 eligible FDG-PET/CT examinations. Fifty-six patients were evaluated with examinations from the Philips Gemini DS scanner and five with examinations from the Philips Gemini TF scanner. The mean FDG-dose was 371 ± 25 (standard deviation [SD]) MBq and the mean uptake time was 67 ± 10 (SD) min.

### Response evaluation

**Figure 1:**
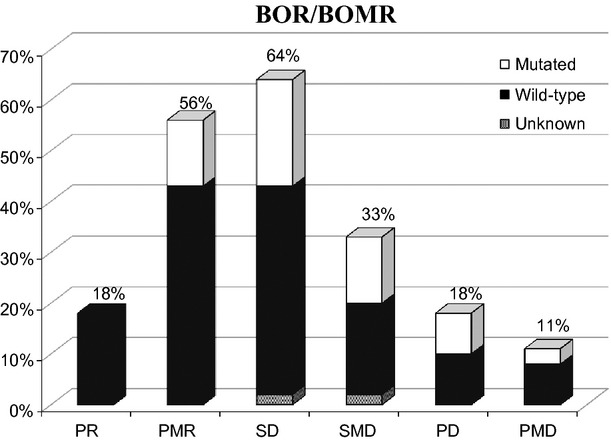
BOR/BOMR according to RECIST and PERCIST. P(M)R, partial (metabolic) response; S(M)D, stable (metabolic) disease; P(M)D, progressive (metabolic) disease; BO(M)R, best overall (metabolic) response.

None of the patients had CR or CMR. According to RECIST, 11 patients (18%) had PR, 39 (64%) had SD, and 11 (18%) had PD as their BOR (Fig.1 and Table 3). The RR was 18%.

According to PERCIST, 34 patients (56%) had PMR, 20 (33%) had SMD, and seven (11%) had PMD as their BOMR (Fig.1 and Table 3). The MRR was 56%. RECIST 1.0 and PERCIST agreed on BOR/BOMR in 28 out of 61 patients (46%). The corresponding *κ*-coefficient was 0.19 and strengths of agreement poor (Table 2). In 35 patients (57%) PD and PMD were coincident. In 11 patients (18%) PMD had occurred earlier than PD was stated and in 15 patients (25%) PMD had not yet occurred at the time of PD.

**Table 2:** Agreement on BOR/BOMR between CT- and FDG-PET/CT-based response evaluations

Response RECIST 1.0					

Response PERCIST	CR	PR	SD	PD	Total PERCIST
CMR	**0**	0	0	0	0
PMR	0	**10**	20	4	34
SMD	0	1	**15**	4	20
PMD	0	0	4	**3**	7
**Total RECIST**	0	11	39	11	**61**

CR, complete response; PR, partial response; SD, stable disease; PD, progressive disease; CMR, complete metabolic response; PMR, partial metabolic response; SMD, stable metabolic disease; PMD, progressive metabolic disease.

**Table 3:** Response categories distribution of all patients and according to *KRAS* status

		RECIST			PERCIST		
			
Patient group	Total no. of patients	PR No. (%)	SD No. (%)	PD No. (%)	PMR No. (%)	SMD No. (%)	PMD No. (%)
**All**	61	11 (18)	39 (64)	11 (18)	34 (56)	20 (33)	7 (11)
***KRAS*** **wt**	42	11 (26)	25 (60)	6 (14)	26 (62)	11 (26)	5 (12)
***KRAS*** **mut**	18	0 (0)	13 (72)	5 (28)	8 (44)	8 (44)	2 (11)

One patient's mutation status was not possible to define.

CR, complete response; PR, partial response; SD, stable disease; PD, progressive disease; CMR, complete metabolic response; PMR, partial metabolic response; SMD, stable metabolic disease; PMD, progressive metabolic disease; wt, wild-type; mut, mutations.

### Correlation with overall survival

**Figure 2:**
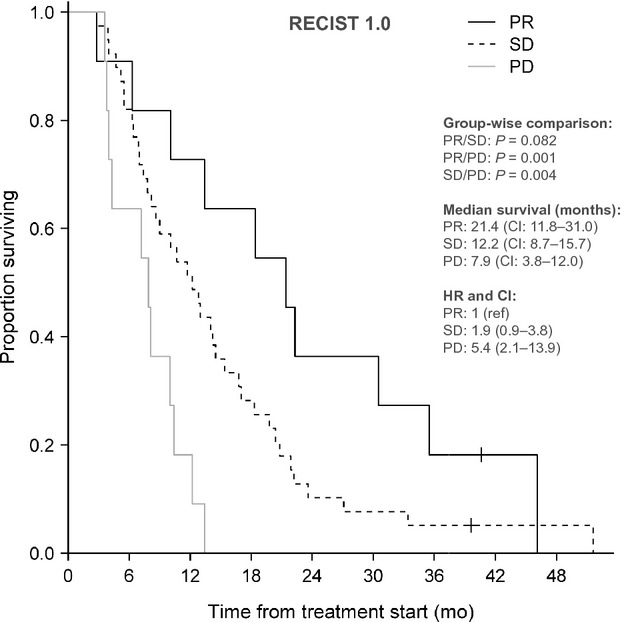
Correlation between survival and response category according to RECIST. PR, partial response; SD, stable disease; PD, progressive disease; HR, hazard ratio; CI, confidence interval.

**Figure 3:**
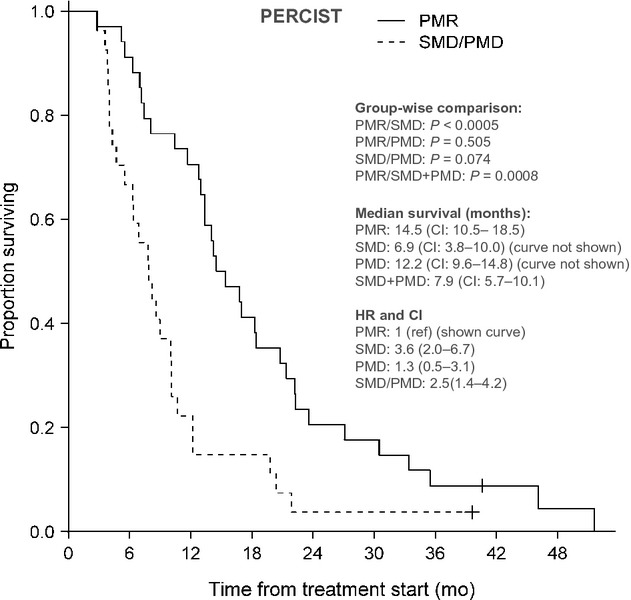
Correlation between survival and response category according to PERCIST. PMR, partial metabolic response; SMD, stable metabolic disease; PMD, progressive metabolic disease; HR, hazard ratio; CI, confidence interval.

On 1st February 2012, two patients were still alive. The correlation between RECIST and PERCIST evaluation and OS as well as group-wise median OS and corresponding 95% CI are shown in Figures2, 3. Survival of the patients in the PR group was not significantly longer than for the patients in the SD group although a trend in this direction was observed (*P* = 0.082, HR = 1.9, CI = 0.9–3.8), but was significantly longer than for the patients in the PD group (*P* = 0.001, HR = 5.4, CI = 2.1–13.9). Due to the low number of patients with PMD (seven), this group was added to the SMD group for the Kaplan–Meier plot of OS. OS of patients with PMR was significantly longer than for patients with SMD (*P* = 0.0005, HR = 3.6, CI = 2.0–6.7) and for patients in the combined SMD + PMD group (*P* = 0.0008, HR = 2.5, CI = 1.4–4.2) but not for patients with PMD (*P* = 0.505, HR = 1.3, CI = 0.5–3.1).

### Correlation with *KRAS*

Forty-two (69%) patients had *KRAS* wild-type and 18 (30%) harbored *KRAS* mutations. One patient's mutation status was not possible to define. None of the patients with PR, 34% of the patients with SD, and 45% of the patients with PD harbored *KRAS* mutations (Fig.1). Twenty-four% of the patients with PMR, 42% of the patients with SMD, and 29% of the patients with PMD had *KRAS* mutations (Fig.1 and Table 3).

## Discussion

We compared CT-based with FDG-PET/CT-based response evaluation of patients with mCRC treated with cetuximab and irinotecan and found morphologic and metabolic response assessment to be incongruent with poor agreement on BOR/BOMR. The PMR rate was threefold higher than the PR rate, and the SMD and PMD rates lower than the SD and PD rates. HR for OS for responders (PR/PMR) versus nonresponders (PD/PMD) was higher for CT- than for FDG-PET/CT evaluation and in general, correlation with survival varied with evaluation method. In contrast to no patients with PR, 24% of the patients with PMR harbored *KRAS* mutations equal to 44% of the total number of patients with *KRAS* mutations.

The RR of irinotecan and cetuximab in 3rd line palliative therapy of mCRC patients has by other study groups been found to be 19–23%, similar to the 18% we found in our study 22–24. In patients with mCRC receiving chemotherapy, primarily in 1st line, Monteil et al. 11 found an MRR of 84% which is somewhat higher than the MRR of 54% in our patient group, presumably due to difference in line of treatment. The low agreement on BOR/BOMR in our study is in line with Monteil et al. 11, who likewise found a considerable fraction of the patients shifting from the SD group to the PMR group when response evaluation shifted from CT- to FGD-PET/CT-based. Additionally, the significant difference in survival between the PMR group and the SMD + PMD group found in our study is consistent with the findings of de Geus-Oei et al. 9,10 who demonstrated a significantly longer median survival of patients with marked reductions in SUV compared to patients without in mCRC and nonsmall cell lung cancer.

An advantage of this study is that all CT examinations were performed as FDG-PET/CT examinations. This allowed comparison of BOR from CT evaluation to BOMR from FDG-PET/CT evaluation. Awareness of imaging modality-dependent differences in response outcome could facilitate choices of optimal imaging modalities for response assessment in clinical trials and in standard clinical care. Our study had several limitations. Treatment was terminated at the time point when PD was established on CT regardless of progression status according to FDG-PET/CT. This hindered comparison of progression-free survival (PFS) from the two evaluation methods and PFS would in this phase II trial have been a more relevant secondary endpoint than OS. Also, to represent the whole colorectal cancer population, the number of included patients in our trial is relatively low. However, with 61 patients, it is one of the largest studies in the field. Furthermore, in order to verify the differences depending on the addition of cetuximab to irinotecan, a control group receiving only chemotherapy would have greatly strengthened the study.

The main response outcome difference was the major shift of patients from SD to PMR, showing that tumor size can remain relatively unchanged while tumor metabolism simultaneously can be markedly reduced. Cetuximab and targeted therapies in general tend to be cytostatic rather than cytotoxic with a somewhat varying effect on tumor size correlated to the target of the therapy 16,25. They halt tumor growth by inhibiting proliferation, angiogenesis, and metastatic spread and by promoting apoptosis 25–27. This reduces tumor metabolism but do not necessarily cause a measurable reduction in tumor size 14,15. Due to this mode of action, objective response assessment results in relatively high SD rates and relatively low PR and CR rates, as seen in our study, which could lead to possible underestimation of drug efficacy 14,15,28–30. FDG-PET/CT visualizes the anatomically attenuation corrected glucose metabolism and thereby depicts changes in tumor-cell metabolism 2,31–33. As targeted therapies are considered to reduce metabolism rather than size, it is suggested that metabolic imaging could be able to depict effect more fulfilling than morphological imaging and thereby perhaps provide more applicable RRs 10,14,34. On the other hand, metabolism could be affected by concomitant medication (i.e., prednisolone). Thus, international consensus on methodology for FDG-PET/CT-based response assessment has not yet been fully implemented which complicates comparison of results between trials. Yet, criteria sets for standardization of methodology have been developed, initially by the EORTC PET study group (1999) 35 and latest with PERCIST (2009) by Wahl et al. 19,36, applied in this study.

RECIST 1.0 and PERCIST response distribution correlated, although differently, with survival. Other study groups have found similar correlations between reduction in FDG-uptake and survival of patients with CRC 9,34,37,38. The HR between the PR and SD group was 1.9 and insignificant but between the PMR and SMD group the HR was 3.6 and significant. On the other hand, the HR between the PR and PD group was 5.4 and significant while it was 1.3 between the PMR and PMD group and insignificant. In other words, the HR for OS for responders (PR/PMR) versus nonresponders (PD/PMD) was higher for CT- than for FDG-PET/CT evaluation. Yet, the study was not powered to draw firm conclusions about survival outcome.

Four patients with SD shifted to PMD. These patients would have had a shorter treatment span if continuation had been based on metabolic tumor changes. However, no patients with PR shifted to PMD, suggesting that patients with a distinct treatment response on CT would theoretically not be deprived treatment if morphologic response assessment was to be replaced with metabolic in future clinical trial settings.

With morphological imaging criteria, RRs of mCRC patients harboring *KRAS* mutations treated with cetuximab have been found significantly lower than for patients without *KRAS* mutations and there has since 2008/2009 been international consensus that these patients should not be treated with cetuximab 17,39,40. In our study, the above mentioned correlation between the CT-based RR and *KRAS* status was retrieved. This was, however, not the case with the FDG-PET/CT-based evaluation as FDG-PET/CT identified a relatively large number of *KRAS* mutation carrying patients who had a metabolic but not a distinct morphologic treatment response. Explanations for this finding could be that FDG-PET/CT, being a more sensitive method than CT, visualizes the tumor changes in the patients where the treatment induces enough reduction in tumor activity to hinder progression but not enough to reduce size. Thereby presumably visualizing the metabolic changes in the patients with SD according to CT evaluation. In the clinic, we continue treatment in patients showing both PR and SD and it would, therefore, be without clinical consequences to replace CT evaluation with FDG-PET/CT for the time being. However, we found that PD and PMD were not coincident in all patients. So in a future trial, where patients were to be randomized between evaluation with either CT or FDG-PET/CT, individual treatment course could to some degree differ depending on the applied evaluation method and might thereby influence treatment duration. Furthermore, it would be interesting to see if and how treatment duration according to evaluation method would influence survival, a question this study is not designed to answer.

Matching a specific drug or regimen to the visualization modality that as optimal as possible depict the expected type of therapy-effect is important to not miss the full potential or overestimate the effect of a drug or regimen and to optimize patient care 5,16. Imaging stratification is an important part of treatment stratification and clinical trial design where also parallel testing of standard and alternative imaging is relevant to develop image stratification toward being cancer type and therapy specific.

## Conclusion

Morphological response assessment by CT, using RECIST 1.0, and metabolic response assessment by FDG-PET/CT, using PERCIST, were incongruent and agreement poor. A large part of the patients shifted from SD with CT evaluation to PMR when evaluated with FDG-PET/CT. RECIST 1.0 and PERCIST response distribution tended to correlate, although differently, with survival. With RECIST 1.0, no patients with PR but a larger fraction of patients with SD had *KRAS* mutations while with PERCIST, a larger fraction of the patients with PMR had *KRAS* mutations.
